# Sympathetic Response to Postural Perturbation in Stance

**DOI:** 10.3389/fnhum.2021.763582

**Published:** 2021-12-10

**Authors:** Taku Kawasaki, Hitoshi Oda, Yasushi Sawaguchi, Hiroshi Kunimura, Koichi Hiraoka

**Affiliations:** ^1^Graduate School of Comprehensive Rehabilitation, Osaka Prefecture University, Habikino, Japan; ^2^College of Health and Human Sciences, Osaka Prefecture University, Habikino, Japan

**Keywords:** sympathetic response, electrodermal response, galvanic skin response, postural control, perturbation, quiet stance

## Abstract

The purpose of the present study was to elucidate whether the sympathetic response to perturbation in stance represents multiple mental responses, whether perturbation-induced fear of fall is one of the mental responses, and whether the sympathetic response is task specific. While healthy humans maintained stance, the support surface of the feet translated in the forward or backward direction. The phasic electrodermal response (EDR), representing the sympathetic response, appeared 1–1.5 s after the support surface translation. Mostly, perturbation-induced EDRs comprised one peak, but some EDRs were comprised of two peaks. The onset latency of the two-peak EDR was much shorter than that of the one-peak EDR. The second peak latency of the two-peak EDR was similar to the peak latency of the one-peak EDR, indicating that the first peak of the two-peak EDR was an additional component preceding the one-peak EDR. This finding supports a view that perturbation-induced EDR in stance sometimes represents multiple mental responses. The amplitude of the EDR had a positive and significant correlation with fear, indicating that perturbation-induced EDR in stance partially represents perturbation-induced fear of fall. The EDR amplitude was dependent on the translation amplitude and direction, indicating that perturbation-induced EDR in stance is a task specific response. The EDR appeared earlier when the participants prepared to answer a question or when the perturbation was self-triggered, indicating that adding cognitive load induces earlier perturbation-induced mental responses.

## Introduction

Sympathetic activity is represented by electrodermal activity (EDA) at the surface of the skin. Fluctuation in EDA induced by discrete stimuli is called phasic electrodermal response (EDR). The EDR is mediated by the rapid fluctuation in eccrine sweat gland activity induced by the liberation of acetylcholine in the sympathetic nervous system (Dawson et al., 2011). The activity of the ventromedial frontal region, right inferior parietal region, and anterior cingulate gyrus are related to the EDR (Tranel and Damasio, 1994).

Internal body state interacts with mental processes such as engagement, attention, cognitive and mental effort, error detection, decision making, or emotion (Critchley et al., 2013). EDA is linked to the control of standing posture (Sibley et al., 2014), according to a previous finding that the electrodermal level (EDL), representing the tonic level of EDA, was correlated with the center of pressure in stance in participants with great anxiety (Maki and Mcllroy, 1996). Accordingly, perturbation-induced EDR in stance may also be linked to the control process of standing posture.

Indeed, previous studies found that perturbation induces EDR in stance as well as in sitting (Sibley et al., 2008, 2009, 2010b). EDR represents multiple mental responses such as the perception of sensation or preparation for action (Khalfa et al., 2002; Dawson et al., 2011; Boucsein, 2012). Perturbation in stance likely induces multiple mental responses such as change in arousal, attention, or expectancy of the perturbation as well as the perception of sensory input, motor response, or perturbation-induced fear of fall (Sibley et al., 2014). Based on this view, EDR induced by the perturbation in stance may sometimes be comprised of multiple peaks representing those multiple mental responses (Hypothesis 1).

Postural threat, which is situation in which safety of the maintenance of the upright posture is threatened, influences postural control in quiet stance. For example, postural threat, induced by standing at an elevated surface height, causes a smaller displacement and greater displacement frequency of the center of pressure (Adkin et al., 2000; Carpenter et al., 2001, 2006; Hauck et al., 2008; Davis et al., 2009), posterior deviation of the center of pressure and center of gravity (Carpenter et al., 2001, 2006; Brown et al., 2006), an increase in stiffness (Carpenter et al., 2001), and a decrease in the soleus H-reflex excitability (Sibley et al., 2007). Moreover, postural threat while standing on a small surface at an elevated height causes greater corticospinal excitability in the trunk muscles compared with standing on a large surface at an elevated height (Tanaka et al., 2013).

It has been well established that emotional states contribute to EDA (Boucsein, 2012). Some pathways from the amygdala, related to fear (Wood et al., 2014), project to the brain stem autonomic output nuclei (Critchley, 2002), contributing to the EDL (Williams et al., 2001; Dawson et al., 2011). Thus, EDA likely represents fear-induced cortical activity during postural task. This view has been supported by several previous studies. Anxiety, balance confidence, and fear were greater when humans maintained a quiet stance on a small surface at an elevated height (Carpenter et al., 2006; Davis et al., 2009; Tanaka et al., 2013). The EDL was higher when humans maintained a quiet stance at an elevated surface height (Brown et al., 2006; Sibley et al., 2007; Hauck et al., 2008; Davis et al., 2009). Fear was greater when humans maintained a quiet stance on a small surface at an elevated height (Davis et al., 2009). Accordingly, the higher EDL when maintaining stance at an elevated surface height observed in the previous studies was derived from height-induced fear.

Perturbation induces instability of the body in stance, causing fear of fall. Thus, perturbation-induced EDR in stance may partially represent perturbation-induced fear of fall. In a previous study by Sibley et al. (2010b), the EDR amplitude in humans, who maintained stance at an elevated surface height, was not significantly different from that at the ground surface level. This finding means that a threatening situation in stance does not influence the perturbation-induced EDR in stance. In spite of that, it has not been elucidated whether perturbation-induced EDR represents fear (Hypothesis 2).

Electrodermal response partially represents preparation for action (Khalfa et al., 2002; Dawson et al., 2011; Boucsein, 2012). Thus, perturbation-induced EDR in stance may represent the preparation process for the perturbation. Expectation of the perturbation influences the perturbation-induced EDR through central set (Horak et al., 1989; Adkin et al., 2000; Sibley et al., 2008). The displacement of the center of pressure induced by the perturbation in stance is smaller when the onset of the perturbation is predictable (Jacobs and Horak, 2007; Jacobs et al., 2008). Time prediction reduces the late response of the ankle muscles to the perturbation in stance (Fujio et al., 2016; Matsuoka et al., 2020). The corticospinal excitability in the tibialis anterior muscle before the perturbation in stance is greater when the onset of the perturbation is predictable (Fujio et al., 2018). Direction prediction reduces the long-latency muscle response to perturbation (Matsuoka et al., 2020). Those findings indicate that time or direction preparation changes the motor response to the perturbation in stance. As shown above, EDL is linked to postural control in stance (Maki and Mcllroy, 1996). Accordingly, EDR may partially represent the time or direction preparation process of the perturbation in stance (Hypothesis 3).

Even considering the previous findings on perturbation-induced EDR in stance (Sibley et al., 2008, 2010a,b), we cannot rule out the possibility that EDR is a byproduct of a non-specific response such as changes in attention or arousal (Sibley et al., 2014). The EDL is used as a parameter indicating arousal (Maki and Whitelaw, 1993; Maki and Mcllroy, 1996), but the EDR does not necessarily represent the mental responses that are the same as those represented by the EDL, because the cortical activity associated with the EDL is different from that associated with EDR (Critchley et al., 2013). There was no significant correlation between the EDL and EDR in postural control task (Sibley et al., 2010b). In addition, it has been shown that arousal is a distinct mediator of postural control (Horslen and Carpenter, 2011).

Previous findings have shown that some mental responses induced by the perturbation in stance are task-dependently modulated. For example, perturbation-induced EDR in sitting was dependent on the velocity of the perturbation (Sibley et al., 2010a). The latency of the muscle response in the tibialis anterior muscle induced by the backward perturbation (i.e., forward translation of the support surface) in stance was shortened by startling auditory stimulus but that induced by the forward perturbation (i.e., backward translation of the support surface) was not (Nonnekes et al., 2013). The effect of the attention was present on the postural response to the forward translation of the support surface, but it was absent for the backward translation of the support surface (Kunimura et al., 2019a,b). Accordingly, we speculate that the perturbation-induced mental responses, represented by the EDR, are task-specific (Hypothesis 4). To prove this hypothesis, we investigated whether perturbation-induced EDR was dependent on the size or direction of the perturbation.

Visual stimulus induces EDR (Dawson et al., 2017). Vision influences the EDL in quiet stance (Davis et al., 2009). The EDL in standing without vision at the edge of a support surface was lower than that in standing with vision (Sibley et al., 2007). Visual attention influences the postural response to the forward translation of the support surface (Kunimura et al., 2019a). These findings indicate that mental response induced by perturbation in quiet stance is influenced by vision. If this view is true, perturbation-induced mental responses, represented by EDR, are dependent on vision (Hypothesis 5).

Taken together, we tested five hypotheses in the present study. EDR induced by the perturbation in stance is sometimes comprised of multiple peaks representing multiple mental responses (Hypothesis 1). Perturbation-induced EDR represents fear (Hypothesis 2). EDR partially represents time or direction preparation process of the perturbation in stance (Hypothesis 3). The perturbation-induced mental responses, represented by the EDR, are task-specific (Hypothesis 4). Mental responses induced by perturbation in quiet stance are dependent on vision (Hypothesis 5).

## Materials and Methods

### Participants

Fourteen healthy humans aged 31.0 ± 8.2 years (11 males and 3 females) participated in experiment 1, and 14 healthy males aged 33.3 ± 7.4 years participated in experiment 2. The sample size was determined based on the sample size of the previous studies that investigated the sympathetic response to the postural perturbation (the number of the participants ranged from 10 to 15) (Sibley et al., 2007, 2008, 2009, 2010a,b). There was no orthopedic or neurological history in these participants. The purpose of the experiments was explained, and written informed consent was obtained from all participants. The experimental design was approved by the Graduate School of Comprehensive Rehabilitation, Osaka Prefecture University Committee on Research Ethics (Approved number: 2018-113).

### Movable Platform

A movable platform (Movable platform, Uchida Denshi, Tokyo) was placed under the participants’ feet in stance. The size of the platform was 45 cm long and 50 cm wide with a height of 7.4 cm. The parameters of the movable platform are shown in Table 1. We used the parameters of the support surface translation that sufficiently induced the body sway in previous studies (Kunimura et al., 2019a,[Bibr B26][Bibr B32]).

**TABLE 1:** Parameters of movable platform.

	Peak acceleration	Time to peak acceleration	Peak velocity	Duration
	
	(m/s^2^)	(ms)	(cm/s)	(ms)
5 cm				
Forward	56	41	122	88
Backward	53	43	122	86
2 cm				
Forward	31	16	56	68
Backward	33	17	56	66

### Measurements

Sensors measuring the electrodermal resistance were placed over the tips of the right index and ring fingers (T.K.K.2701, Takei Kiki, Tokyo). The anterior–posterior displacement of the pelvis was measured using an acceleration sensor (AS-10TB, Kyowa Dengyo, Tokyo) during experiment 2. This acceleration sensor was placed over the skin at the midpoint between the left and right posterior superior iliac spines with an elastic tape (Kunimura et al., 2019a,b). The signal from the acceleration sensor was amplified with a high-cut filter of 1 kHz using a strain amplifier (CDV-700A; Kyowa Dengyo, Tokyo). Measuring the acceleration of a body part is a valid and reliable method of estimating motor responses during the postural task (Kavanagh and Menz, 2008). Previous studies have shown that the displacement of the trunk in quiet stance or during step initiation was successfully measured by the acceleration sensors placed on C7 and L5 (Mancini et al., 2009; Mancini and Horak, 2010). The analog signals from the acceleration sensor and from the sensors measuring the EDL were converted to digital signals at a sampling rate of 1 kHz using an A/D converter (PowerLab 800S; AD Instruments, Colorado Springs, CO, United States), and the digitized signals were stored on a PC.

### Postural Perturbation Task

The participants maintained a standing position on the movable platform. The distance between the toes was 5 cm. A vertical plain wall was placed 70 cm in front of the participants. An experimenter visually monitored the fluctuation of the EDL. The translation of the support surface was triggered when the EDL did not fluctuate for 3-5 s in the trials with the externally triggered translation. After completion of the data acquisition of the EDR induced by the translation in each trial, the experimenter asked the participants to get off the platform. The participants maintained an upright stance on the platform until the experimenter asked them to get off the platform. Then, another experimenter moved the support surface back to the starting position, and the participants stepped onto the support surface and maintained an upright stance for the next trial. It is recommended that the interval between the stimuli must be no shorter than 20 s so that the EDL can recover to a readiness state at this time (Khalfa et al., 2002). In a previous study measuring EDR after postural perturbation in stance, the trial-to-trial interval was 15–60 s (Sibley et al., 2008). Based on the above, in the present study, the inter-trial interval was 40 s or longer.

### Procedure (Experiment 1)

While the participants closed their eyes, the support surface translated forward with a 5 cm amplitude. Before beginning this experiment, the participants were informed that the forward translation of the support surface would begin without warning. After each translation, they verbally provided answers regarding their magnitude of fear during the translation of the support surface based on the verbal analogue scale (VAS); 0 points indicated no fear, and 100 points indicated maximum fear (Teachman et al., 2008). The number of trials was 12.

### Procedure (Experiment 2)

**FIGURE 1:**
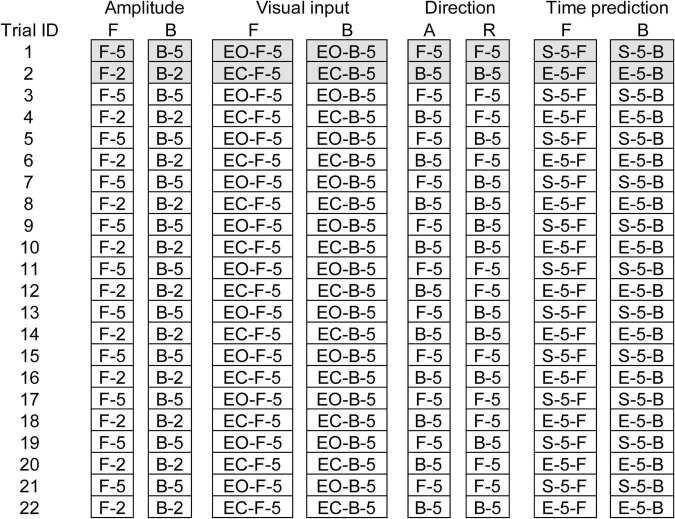
Schedule of eight trial blocks in experiment 2. Filled cells represent trials excluded from the data analysis. A, Alternative direction; R, random direction; F, forward translation; B, backward translation; EO, eyes opened; EC, eyes closed; 2, 2 cm amplitude; 5, 5 cm amplitude.

Eight trial blocks were conducted in experiment 2. In each block, a comparison was made between the two conditions as shown in Table 2. The effect of the translation amplitude was tested in a trial block with the forward translation and in another trial block with the backward translation. While the participants closed their eyes, a trial with the support surface translation with a 2 cm amplitude and another with the translation with a 5 cm amplitude were given alternatively in each trial block. This trial pair (i.e., a trial with a 2 cm amplitude translation and another trial with a 5 cm amplitude translation) was repeated 11 times (22 trials in each block in total) (Figure 1).

**TABLE 2:** Trial blocks in Experiment 2.

Effect tested	Trial block	Condition
Amplitude	Forward	5 cm
		2 cm
	Backward	5 cm
		2 cm
Direction (random)	5 cm	Forward
		Backward
Direction (alternative)	5 cm	Forward
		Backward
Vision	Forward	Eyes-opened
		Eyes-closed
	Backward	Eyes-opened
		Eyes-closed
Time prediction	Forward	Self trigger
		External trigger
	Backward	Self trigger
		External trigger

The effect of vision was tested in a trial block with the forward translation and in another trial block with the backward translation (Table 2). The amplitude of the translation was 5 cm. In each block, a trial with the eyes opened and another trial with the eyes closed were given alternatively (a pair of trials), and this pair of trials was repeated 11 times (22 trials in total) (Figure 1).

The effect of time prediction was tested in a trial block with the forward translation and another trial block with the backward translation (Table 2). The participants closed their eyes in each trial. A touch sensor device triggering the translation (trigger device) was attached over the tip of the left index finger. In a trial with self-triggered translation, the participants triggered the translation of the support surface at their own preferred time by tapping the trigger device with the left thumb. In a trial with externally triggered translation, an experimenter triggered it without warning. Two conditions (i.e., self-triggered and externally triggered translations) were alternatively given for a trial pair, and this pair was repeated 11 times (22 trials) in each block (Figure 1). The order of the two conditions in each trial block testing the effect of the translation amplitude, vision, or time prediction was counterbalanced across the participants, and participants were informed before the beginning the trial block.

Two trial blocks, alternative and random blocks, were conducted to test the effect of the translation direction (Table 2). The amplitude of the translation was 5 cm. The participants closed their eyes. Twenty-two trials, consisting of eleven trials with the forward translation and eleven trials with backward translation, were conducted in each block (Figure 1). In the first two trials, one was forward translation, and the other was the backward translation in both the random and alternative blocks.

In the random block, the direction of the platform translation was randomly altered trial by trial. Because the direction of the translation was given in a random order, the participants could not predict the direction of the forthcoming translation in the random block. In the alternative block, the direction of the translation was alternatively assigned to each trial. The order of the two directions assigned (i.e., forward translation first or backward translation first) was counterbalanced across the participants. Before beginning each task, the participants were informed of the translation direction. Thus, the participants could predict the direction of the forthcoming translation in the alternative block.

### Data Analysis

Electrodermal response is significantly influenced by the novelty of the stimulus (Sibley et al., 2008). Thus, the first two trials in each block were excluded from the data analysis, in accordance with a recommendation in a previous study (Khalfa et al., 2002) as well as with a previous finding that the EDR amplitude tended to be large in the first several trials (Sibley et al., 2008). The fluctuation of the EDL continuing 2 s or more in the time window between 2 s before the translation of the support surface and 6 s after that was considered to be the EDR. The base to peak amplitude of the EDR and the onset and peak latencies were calculated. The baseline was the average EDL in the time window 0–100 ms before the translation onset in the trials in which the EDR appeared after the translation onset. In the trials in which the EDR appeared before the translation onset, the baseline was measured in the time window 0–100 ms before the EDR onset. Traces indicating the anterior–posterior acceleration of the pelvis were integrated twice to estimate the anterior–posterior displacement. The base to peak amplitude of pelvis displacement and the onset latency were calculated. The baseline of the pelvis displacement was measured in the time window 0–100 ms before the translation onset.

Two-way repeated-measures ANOVA was conducted to test the two main effects (i.e., translation direction [forward and backward translation] and task [5 cm, 2 cm, eyes opened, eyes closed, random direction, alternative direction, external trigger, and self trigger]). The result of Greenhouse–Geisser’s correction was reported whenever Mauchly’s test of sphericity was significant. A paired *t*-test was conducted for testing the difference between the two means. The Pearson’s correlation coefficient between the EDR amplitude or onset latency and the VAS indicating fear was calculated in each participant. A one-sample *t*-test was conducted to test whether the mean correlation coefficient across the participants was different from zero. For the comparison between the mean in experiment 1 and that in experiment 2, an unpaired *t*-test was conducted. For the subgroup analysis for the amplitude and latency of the one-peak and two-peak EDRs, a Mann–Whitney *U*-test was conducted. The alpha level was 0.05. Excel–Toukei 2010 v1.13 (Social Survey Research Information, Tokyo) was used for the statistical analysis. The data are expressed as the mean and standard error of the mean.

## Results

### Electrodermal Response and Fear in Experiment 1

**FIGURE 2:**
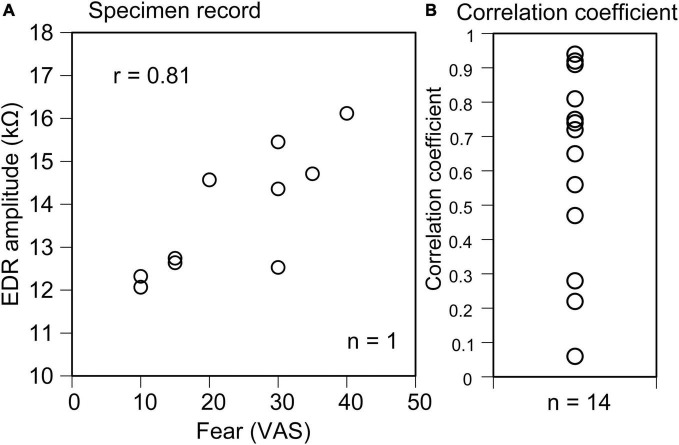
Scatter plot of the EDR amplitude as the function of the VAS indicating fear in a participant **(A)** and plot of the correlation coefficient between the EDR amplitude and VAS indicating fear across the participants **(B)**.

In all trials across participants, EDR appeared after the translation of the support surface in Experiment 1. The average onset latency was 1.10 ± 0.12 s, and the average amplitude was 11.2 ± 2.2 kΩ. In some trials, two-peak EDRs appeared with a probability of 0.29 ± 0.08. The average score of the VAS, representing fear induced by the support surface translation across the participants, was 38.1 ± 6.7. The scatter plot of the EDR amplitude as the function of the VAS in a participant is shown in Figure 2A. The correlation coefficient was positive across all participants (Figure 2B). The mean correlation coefficient between the EDR amplitude and VAS was 0.58 ± 0.08, and it was significantly greater than zero (*p* < 0.001, one-sample *t*-test). The mean correlation coefficient between the EDR onset latency and VAS was not significantly different from zero.

### Electrodermal Response in Experiment 2

The EDR was successfully induced by the translation of the support surface in all trials across the participants. The onset latency of the EDR was approximately 1.5 s except for the trials with the self-triggered translation (Tables 3–6). In the Experiment 1, the externally triggered forward translation with a 5 cm amplitude was given. To compare the EDR in the Experiment 2 with that in the Experiment 1, the EDRs induced by the perturbation same parameters as the Experiment 1 (externally triggered forward translation with a 5 cm amplitude) were averaged across the trial blocks in the Experiment 2. Supposedly, the onset latency of the EDR must have been similar between the experiments, because of the same perturbation parameters between those. In spite of this supposition, the onset latency of the EDR in Experiment 2 was significantly longer than that in Experiment 1 (*p* = 0.048, unpaired *t*-test).

**TABLE 3:** Effect of time prediction.

	EDR					Pelvis displacement				
	Self trigger		External trigger			Self trigger		External trigger		
Amplitude										
Forward	11.1	(1.2)	9.7	(0.9)		1.10	(0.08)	1.27	(0.10)	*
Backward	11.5	(1.6)	10.3	(1.8)		0.76	(0.06)	0.77	(0.06)	
Onset latency										
Forward	0.64	(0.23)	1.56	(0.11)	*	38.07	(1.34)	37.79	(1.25)	
Backward	0.52	(0.29)	1.43	(0.11)	*	38.07	(1.55)	37.00	(1.26)	

Unit: EDR amplitude (kΩ); EDR latency (s); Pelvis displacement amplitude (cm); Pelvis displacement latency (ms); *p < 0.05 (self vs. externally triggered), Mean (standard error of mean).

**TABLE 4:** Effect of translation amplitude.

	EDR					Pelvis displacement				
	5 cm		2 cm			5 cm		2 cm		
Amplitude										
Forward	12.6	(1.3)	5.8	(1.1)	*	1.40	(0.10)	0.40	(0.03)	*
Backward	10.5	(1.4)	4.7	(0.8)	*	0.79	(0.05)	0.33	(0.02)	*
Onset latency										
Forward	1.61	(0.13)	1.75	(0.11)		38.50	(1.36)	34.80	(0.97)	*
Backward	1.55	(0.13)	1.80	(0.10)	*	35.93	(0.84)	33.28	(0.78)	*

Unit: EDR amplitude (kΩ); EDR latency (s); Pelvis displacement amplitude (cm); Pelvis displacement latency (ms); *p < 0.05 (5 cm vs. 2 cm), Mean (standard error of mean).

**TABLE 5:** Effect of translation direction.

	EDR					Pelvis displacement				
	forward		backward			forward		backward		
Amplitude										
Alternative	12.2	(1.2)	9.5	(1.4)	*	1.41	(0.11)	0.79	(0.05)	*
Random	13.4	(1.6)	10.3	(1.3)	*	1.41	(0.11)	0.75	(0.06)	*
Onset latency										
Alternative	1.56	(0.14)	1.54	(0.13)		38.36	(1.79)	36.43	(0.78)	
Random	1.56	(0.12)	1.36	(0.15)		38.07	(2.02)	35.71	(0.99)	

Unit: EDR amplitude (kΩ); EDR latency (s); Pelvis displacement amplitude (cm); Pelvis displacement latency (ms); *p < 0.05 (forward vs. backward), Mean (standard error of mean).

**TABLE 6:** Effect of vision.

	EDR				Pelvis displacement				
	Eyes opened		Eyes closed		Eyes opened		Eyes closed		
Amplitude									
Forward	10.0	(0.9)	9.8	(1.1)	1.43	(0.12)	1.34	(0.10)	*
Backward	10.0	(1.6)	9.6	(1.7)	0.77	(0.06)	0.74	(0.06)	
Onset latency									
Forward	1.64	(0.10)	1.52	(0.15)	37.66	(1.29)	37.50	(1.46)	
Backward	1.54	(0.10)	1.52	(0.15)	35.14	(0.85)	36.21	(0.89)	

Unit: EDR amplitude (kΩ); EDR latency (s); Pelvis displacement amplitude (cm); Pelvis displacement latency (ms); *p < 0.05 (eyes opened vs. closed), Mean (standard error of mean).

### Effect of Time Prediction

**FIGURE 3:**
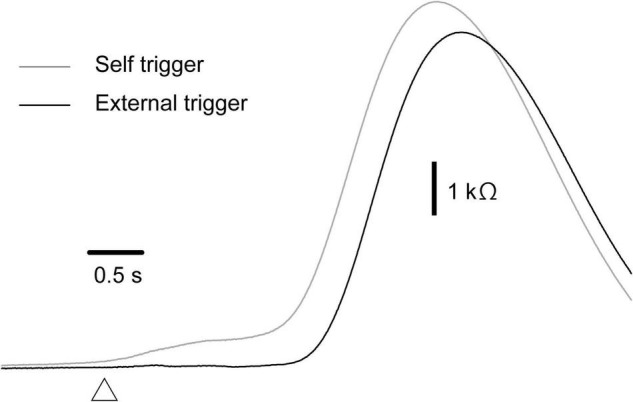
Averaged traces of EDR across 10 trials in the block testing the effect of the time prediction in a participant.

The effect of time prediction is shown in Table 3. Typical traces of the EDRs after the self-triggered and externally triggered perturbation in a participant are shown in Figure 3. On the one hand, there was no significant difference in the EDR amplitude between the self-triggered and externally triggered translation. On the other hand, the EDR onset latency with the self-triggered translation was significantly shorter than that with the externally triggered translation both in the forward (*p* < 0.001, *t*-test) and backward translation blocks (*p* = 0.004, *t*-test). Pelvis displacement after the self-triggered forward translation was significantly smaller than that after the externally triggered forward translation (*p* = 0.005, *t*-test).

### Effect of Translation Amplitude

**FIGURE 4:**
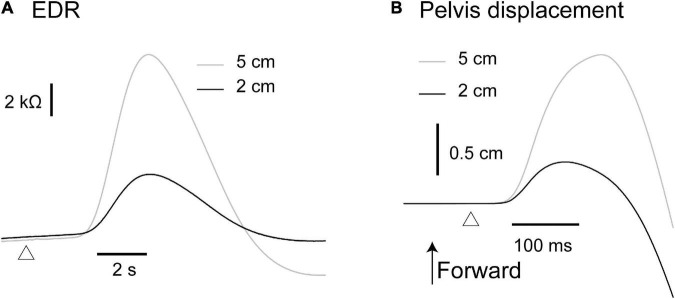
Averaged traces of the EDR **(A)** and pelvis displacement **(B)** across 10 trials in the block testing the effect of the translation amplitude in a participant.

The effect of the translation amplitude is shown in Table 4. The averaged traces of the EDR and pelvis displacement after the forward translation with a 5 cm amplitude in a participant are shown in Figure 4. The amplitude of the EDR induced by the translation with a 5 cm amplitude was significantly greater than that with a 2 cm amplitude, both in the forward (*p* < 0.001, *t*-test) and backward translation blocks (*p* < 0.001, *t*-test). Similarly, the pelvis displacement induced by the translation with a 5 cm amplitude was significantly greater than that with a 2 cm amplitude, both in the forward (*p* < 0.001, *t*-test) and backward translation blocks (*p* < 0.001, *t*-test).

The onset latency of the EDR after the backward translation with a 2 cm amplitude was significantly longer than that with a 5 cm amplitude (*p* = 0.030, *t*-test). For the forward translation, the tendency was similar to the backward translation (*p* = 0.082, *t*-test). The onset latency of the pelvis displacement induced by the translation with a 5 cm amplitude was significantly longer than that with a 2 cm amplitude, both in the forward (*p* < 0.001, *t*-test) and backward translation blocks (*p* < 0.001, *t*-test).

### Effect of Translation Direction

**FIGURE 5:**
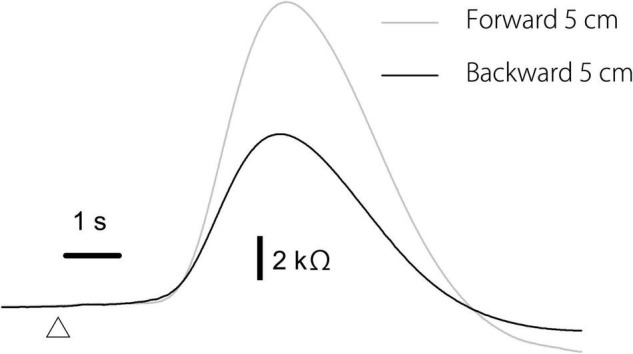
Averaged traces of the EDR across 10 trials in the block testing the effect of the translation direction in a participant.

The effect of the translation direction is shown in Table 5. Averaged traces of the EDR after the forward and backward translation of the support surface in a participant are shown in Figure 5. The amplitude of the EDR induced by the forward translation was significantly greater than that induced by the backward translation both in the alternative (*p* = 0.031, *t*-test) and random blocks (*p* = 0.020, *t*-test). Similarly, the amplitude of the pelvis displacement induced by the forward translation was significantly greater than that induced by the backward translation both in the alternative and random blocks (*p* < 0.001, *t*-test). There was no significant difference in the onset latency of the EDR and pelvis displacement between the forward and backward translation conditions.

### Effect of Vision

The effect of vision is shown in Table 6. There was no significant difference in the amplitude and onset latency of the EDR between the eyes-opened and eyes-closed conditions, in both the forward and backward translation blocks. The pelvis displacement induced by the forward translation of the support surface with the eyes opened was significantly greater than that with the eyes closed (*p* = 0.008, *t*-test).

### One- and Two-Peak Electrodermal Response

**FIGURE 6:**
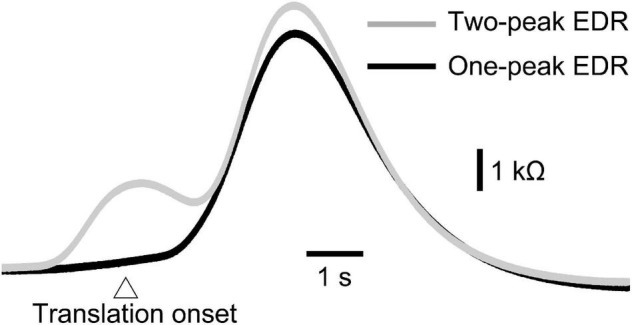
Averaged one-peak and two-peak EDR induced by the self-triggered translation of the support surface across 10 trials in a participant.

As with experiment 1, two-peak EDR appeared in Experiment 2. Traces of one- and two-peak EDR in a participant are shown in Figure 6. The appearance probability of two-peak EDR after translation with a 5 cm amplitude across the participants in Experiment 2 is shown in Table 7. There was no significant main effect of the translation direction (*F*(1,13) = 0.799, *p* = 0.388), but there was a significant main effect of the task (*F*(7,91) = 2.618, *p* = 0.017) on the appearance probability. There was no significant interaction between the main effects (*F*(15.28,187.41) = 1.060, *p* = 0.385) (Greenhouse–Geisser correction; Mauchly’s test *p* < 0.05). The appearance probability of two-peak EDR for the forward translation with a 5 cm amplitude across tasks was 0.18 ± 0.05. This probability tended to be lower than that in experiment 1, but the difference was insignificant (*p* = 0.160, Mann–Whitney *U*-test).

**TABLE 7:** Appearance probability of two-peak EDR.

	Forward		Backward	
Amplitude (5 cm)	0.14	(0.07)	0.18	(0.07)
Amplitude (2 cm)	0.06	(0.03)	0.04	(0.02)
Eyes opened	0.14	(0.05)	0.13	(0.06)
Eyes closed	0.24	(0.08)	0.18	(0.08)
Direction (a)	0.15	(0.08)	0.17	(0.07)
Direction (r)	0.15	(0.07)	0.24	(0.08)
External trigger	0.14	(0.06)	0.24	(0.08)
Self trigger	0.24	(0.06)	0.30	(0.07)

*a, alternative; r, random; Mean (standard error of mean).*

The onset latency of the one-peak EDR and that of the two-peak EDR are shown in Table 8. The onset latency of the two-peak EDR was significantly shorter than that of the one-peak EDR across the tasks (*p* < 0.05; Mann–Whitney *U*-test, indicated by asterisks in Table 8). The onset latency of one- or two-peak EDR with the externally triggered translation was significantly longer than that with the self-triggered translation (*p* < 0.05; Mann–Whitney *U*-test, indicated by daggers in Table 8). Interestingly, the onset latency of two-peak EDR appeared 1 s or more before the onset of the self-triggered translation in all participants. The onset latency of two-peak EDR with a 5 cm translation amplitude was significantly longer than that with a 2 cm translation amplitude (*p* < 0.05; Mann–Whitney *U*-test, indicated by daggers in Table 8). In the random block for testing the effect of the translation direction, the onset latency of the two-peak EDR induced by the backward translation was significantly shorter than that induced by the forward translation (*p* = 0.002, Mann–Whitney *U*-test, indicated by a double dagger in Table 8).

**TABLE 8:** Onset latency of one- and two-peak EDR.

	Forward (s)							Backward (s)						
	One peak			Two peaks				One peak			Two peaks			
Amplitude (5 cm)	1.82	(0.07)		0.33	(0.04)		*	1.83	(0.09)		0.38	(0.20)		*
						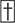							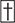	
Amplitude (2 cm)	1.86	(0.09)		–0.11	(0.23)		*	1.87	(0.09)		–0.15	(0.20)		*
Eyes opened	1.88	(0.08)		0.15	(0.12)		*	1.74	(0.08)		0.12	(0.12)		*
Eyes closed	1.89	(0.07)		0.31	(0.06)		*	1.79	(0.07)		0.21	(0.04)		*
Direction (a)	1.79	(0.06)		0.52	(0.27)		*	1.83	(0.07)		0.21	(0.06)		*
Direction (r)	1.79	(0.05)		0.33	(0.05)		*	1.78	(0.06)		–0.01	(0.08)	‡	*
External trigger	1.78	(0.07)		0.22	(0.09)		*	1.71	(0.07)		0.18	(0.03)		*
			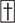			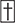				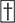			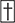	
Self trigger	1.04	(0.14)		–1.46	(0.39)		*	0.86	(0.20)		–1.21	(0.24)		*

*p < 0.05 (one- vs. two-peak); ^†^p < 0.05 (between tasks); ^‡^p < 0.05 (Forward vs. backward in two-peak EDR); Mean (standard error of mean); a, alternative; r, random.

The peak latency of the one-peak EDR and the second peak latency of the two-peak EDR are shown in Table 9. The peak latency of the one-peak EDR and the second peak latency of the two-peak EDR were similar across all tasks. There was no significant difference between the peak latency of the one-peak EDR and the second peak latency of the two-peak EDR. The amplitude of the one-peak EDR and that of the second peak of the two-peak EDR are shown in Table 10. There was no significant difference in the amplitude between the one-peak EDR and the second peak of the two-peak EDR.

**TABLE 9:** Peak latency of one-peak EDR and that of second peak in two-peak EDR.

	Forward (s)				Backward (s)			
	One peak		Two peaks		One peak		Two peaks	
Amplitude (5 cm)	3.7	(0.2)	3.7	(0.2)	3.6	(0.2)	4.0	(0.3)
Amplitude (2 cm)	3.6	(0.2)	3.4	(0.1)	3.5	(0.1)	3.2	(0.1)
Eyes opened	3.7	(0.1)	3.6	(0.2)	3.5	(0.1)	3.8	(0.3)
Eyes closed	3.7	(0.1)	3.7	(0.2)	3.5	(0.2)	3.3	(0.1)
Direction (a)	3.6	(0.1)	3.8	(0.2)	3.6	(0.1)	3.6	(0.2)
Direction (r)	3.6	(0.1)	3.7	(0.2)	3.6	(0.1)	3.5	(0.1)
External trigger	3.7	(0.1)	3.7	(0.2)	3.6	(0.1)	3.6	(0.1)
Self trigger	3.3	(0.2)	3.2	(0.1)	3.3	(0.1)	3.0	(0.1)

*a, alternative; r, random, Mean (standard error of mean).*

**TABLE 10:** Amplitude of one-peak EDR and that of second peak in two-peak EDR.

	Forward (kΩ)				Backward (kΩ)			
	One peak		Two peaks		One peak		Two peaks	
Amplitude (5 cm)	11.8	(1.7)	12.5	(2.5)	10.6	(1.4)	9.3	(1.5)
Amplitude (2 cm)	6.0	(1.1)	6.2	(2.1)	4.6	(0.8)	7.7	(3.0)
Eyes opened	9.9	(0.8)	11.0	(0.9)	9.4	(1.5)	9.1	(1.5)
Eyes closed	9.7	(1.1)	8.2	(1.3)	9.0	(1.5)	10.4	(2.9)
Direction (a)	11.4	(1.6)	10.2	(2.7)	8.8	(1.1)	11.0	(2.4)
Direction (r)	13.8	(1.7)	12.8	(1.5)	10.1	(1.3)	7.9	(1.5)
External trigger	9.4	(0.8)	10.3	(1.2)	9.9	(1.7)	8.1	(1.5)
Self trigger	10.9	(1.5)	9.2	(1.2)	11.6	(1.6)	11.7	(2.0)

*a, alternative; r, random, Mean (standard error of mean).*

## Additional Experiment

In experiment 2, two-peak EDR appeared before the self-triggered translation. The motor process for the finger-tapping movement must occur before the self-triggered translation. Thus, we hypothesized that the first component of EDR that appears before the self-triggered translation is induced by the motor process of the finger-tapping movement. To test this hypothesis, an additional experiment, testing whether the EDR appears before the finger-tapping movement in stance, was conducted.

**FIGURE 7:**
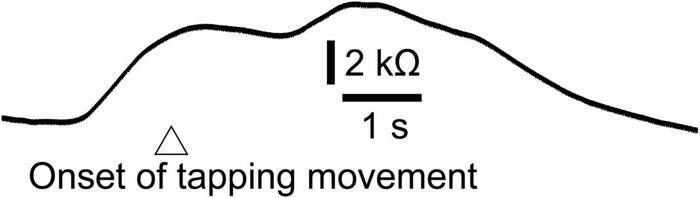
Averaged trace of the EDR induced by the finger-tapping movement across 10 trials in a participant.

Twelve male participants aged 32.4 ± 8.5 years tapped a trigger device, placed over the tip of the left index finger, with the left thumb at their own preferred time in stance. The number of trials was 12, and the first two trials were excluded from the data analysis. The EDR appeared before the onset of the finger-tapping movement. The averaged trace of the EDR is shown in Figure 7. The onset latency and appearance probability of this EDR induced by the finger-tapping movement were similar to the two-peak EDR in the trials with the self-triggered translation in Experiment 2 (Table 11). The amplitude, onset latency, and appearance probability of the EDR induced by the finger-tapping movement were not significantly different from those of the two-peak EDR in the trials with the self-triggered translation in Experiment 2.

**TABLE 11:** EDR induced by self-triggered translation and tapping-triggered EDR.

	Self-triggered		Tapping-triggered	
Appearing probability	0.21	(0.06)	0.26	(0.06)
Onset latency (s)	–1.56	(0.33)	–1.45	(0.19)
Amplitude (kΩ)	6.37	(1.15)	3.71	(0.88)

*Mean (standard error of mean).*

## Discussion

### Perturbation-Induced Electrodermal Response in Stance

As consistent with previous studies (Sibley et al., 2008, 2010b), EDR was induced by postural perturbation in stance. EDR is induced by various discrete stimuli (Dawson et al., 2017). Our present findings indicate that perturbation in stance is one of those discrete stimuli inducing EDR. The appearance probability of EDR after the perturbation in stance was 1.00. This finding was inconsistent with a previous finding that EDR was present in 65% of the trials in which the onset of the perturbation was predictable, and in 97% of the trials in which the onset of the perturbation was not predictable (Sibley et al., 2008). In the present study, perturbation was induced by the support surface translation, but it was induced by the perturbation over the trunk in the previous study. Such a difference in the target body part of the perturbation may be the reason for the difference in the appearance probability of the EDR between those studies. The response of the ankle is predominant for the support surface translation when the support surface is large (Horak and Nashner, 1986). The proximal body parts must response to the trunk perturbation. Thus, difference in the part of the body (ankle or trunk) that mainly responds to the perturbation may be the cause of the difference in the appearance probability of the EDR between the studies.

### Multiple Mental Responses

The EDR represents multiple mental responses such as perception of sensation or preparation for action (Khalfa et al., 2002; Dawson et al., 2011; Boucsein, 2012). Multiple mental responses, such as perception of sensation induced by the body inclination and preparatory activity for the response to the perturbation, must be processed during the perturbation in stance. Based on this view, the EDR induced by the perturbation in stance must sometimes be comprised of multiple fluctuations in EDA representing multiple mental responses (Hypothesis 1).

Consistent with this hypothesis, two-peak EDRs were sometimes induced by perturbation in stance. Although morphological observation of the monophasic and diphasic fluctuations in the EDA without stimulation has been conducted in a previous study (de Bonis and Baqué, 1978), the present finding is the first to show that two-peak EDRs are sometimes observable after perturbation in stance. On the one hand, the onset latency of the two-peak EDR was shorter than that of the one-peak EDR. On the other hand, there was neither significant difference in the peak latency between the one-peak EDR and the second peak of the two-peak EDR nor significant difference in the amplitude between those, indicating that the second peak of the two-peak EDR was the same component as the peak of the one-peak EDR. Based on this, the first peak of the two-peak EDR must be an additional component preceding the one-peak EDR. This means that early and late peaks of the two-peak EDR represent multiple mental responses.

The finding regarding the self-triggered perturbation-induced EDR is indirect evidence indicating the multiple mental responses contributing to the two-peak EDR. On the one hand, the onset of the one-peak EDR was always later than the perturbation onset. This means that the one-peak EDR was induced by the perturbation. On the other hand, the onset of the two-peak EDR induced by the self-triggered perturbation was earlier than the perturbation onset. Thus, the first component of the two-peak EDR induced by the self-triggered perturbation represents the mental response before the perturbation. The EDR is dependent on the muscle activity level (Dimberg, 1990), indicating that the motor process is one mental process represented by the EDR. In the additional experiment in our present study, EDR was induced by finger-tapping movement without perturbation. The perturbation in stance induced the EDR, and the onset latency of this EDR was similar to that of the two-peak EDR induced by the self-triggered perturbation. Thus, the first component of the two-peak EDR, onsets before the self-triggered perturbation, represents the execution process of the finger-tapping movement. Taken together, the self-triggered perturbation-induced EDR was comprised of both the motor execution process before the perturbation and the mental responses induced by the perturbation.

### Perturbation-Induced Fear

The amygdala, related to fear, contributes to EDR (Williams et al., 2001; Dawson et al., 2011). Fear is an appropriate stimulus inducing the EDR (Öhman and Mineka, 2001; Dawson et al., 2017). Perturbation in stance causes instability of the posture and, thus, likely induces fear. Accordingly, we hypothesized that EDR partially represents perturbation-induced fear of fall (Hypothesis 2). A significant positive correlation was found between the EDR amplitude and fear. This indicates that trial-to-trial variation of the EDR is partly derived from the trial-to-trial variation of fear. This finding supported Hypothesis 2.

### Preparation Process

The EDR represents recognition of the preparation process (Dawson et al., 2017). Expectation of the perturbation influences the perturbation-induced EDR through central set (Horak et al., 1989; Adkin et al., 2000; Sibley et al., 2008). Some predictions, time or direction prediction in the present study, induce expectation, and preparation process, that changes central set, occurs based on expectation. Thus, perturbation-induced EDR may partially represent preparation process for the response to the perturbation (Hypothesis 3). The post-perturbation N1 potentials after the externally triggered perturbation were greater than that after the self-triggered perturbation (Mochizuki et al., 2009). This finding indicates that the cortical response to the perturbation is greater after the externally triggered perturbation. Based on this previous finding, one may speculate that the mental response induced by the perturbation may be greater when the perturbation is externally triggered. In line with this view, a previous study has shown that time prediction decreases the perturbation-induced EDR amplitude in stance (Sibley et al., 2008).

In the present study, such a significant change in the perturbation-induced EDR amplitude induced by time prediction was not found and thus Hypothesis 3 was not supported. The study design for testing time prediction in the present study was the same as that in a previous study (Sibley et al., 2008); testing the effect of time prediction was achieved through comparing EDR induced by the self-generated perturbation and that induced by the externally triggered perturbation. One major difference between the two studies is visual input; the eyes were opened in the previous study, but the eyes were closed in the present study. Thus, a possible explanation for the different findings between the previous and present studies is that perturbation-induced EDR is enhanced by adding a time preparation process only when vision is present.

### Task-Specific Response

It has been stated that we cannot rule out the possibility that perturbation-induced EDR is a byproduct of non-specific mental responses such as changes in attention or arousal (Sibley et al., 2014). The EDR amplitude after the support surface translation with a 5 cm amplitude was significantly greater than that after the translation with a 2 cm amplitude. This finding was consistent with a previous finding that the perturbation-induced EDR in sitting was dependent on the velocity of the perturbation (Sibley et al., 2010a). Moreover, the amplitude of the EDR induced by the forward translation of the support surface was significantly greater than that induced by the backward translation in the present study. These present findings supported Hypothesis 4: perturbation-induced EDR in stance is a task-specific response.

One alternative explanation for the finding that EDR is dependent on the perturbation size or direction is the intensity of the stimulus inducing the EDR. The motor response to the perturbation in stance is dependent on the intensity of the perturbation (Diener et al., 1984, 1988). More importantly, EDR is greater with the increase in the intensity of the stimulus inducing the EDR (Dimberg, 1990; Turpin et al., 1999; Dawson et al., 2017). This means that the size of the EDR is dependent on the intensity of sensory input produced by the stimulus. The pelvis displacement after the support surface translation with a 5 cm amplitude was greater than that after the translation with a 2 cm amplitude in the present study. In addition, pelvis displacement induced by the forward translation was significantly greater than that induced by the backward translation. Greater pelvis displacement reflects greater body sway, producing greater sensory input. Thus, the effect of the perturbation size and direction on the EDR size observed in the present study is explained by a view that greater perturbation-induced body sway producing more intensive sensory input causes greater EDR.

Particularly regarding the dependency of the EDR on the translation direction, an alternative explanation is available. Humans grasp the toes to recover the body from the forward inclination induced by the backward translation of the support surface, but such response does not work with the forward translation. Thus, safety margin of the response to the backward translation is greater than that to the forward translation. This means that the forward translation of the support surface must be more fearful than the backward translation. Thus, the difference in safety margin between the forward and backward translation, causing direction-dependent fear, may be a possible explanation regarding the dependency of the EDR on the translation direction.

### Cognitive Load-Induced Earlier Electrodermal Response

The onset latency of the EDR induced by the self-triggered perturbation was shorter than that induced by the externally triggered translation. The onset of the perturbation was predictable when the perturbation was triggered by the participants themselves, but was not predictable when it was triggered by an experimenter. Thus, our finding indicates that time preparation causes the earlier mental responses induced by the perturbation in stance.

The onset latency of the EDR in Experiment 2 was significantly longer than that in Experiment 1. In Experiment 1, the participants were asked to answer the extent of the perturbation-induced fear after the perturbation. This means that a cognitive task was assigned to the participants in Experiment 1. In contrast, in the externally triggered perturbation in Experiment 2, such a cognitive task was not assigned. Accordingly, the earlier onset of the EDR in Experiment 1 was likely caused by adding cognitive load. Taken together, the earlier perturbation-induced EDR in stance observed in the self-triggered perturbation and in the trials of Experiment 1 was likely caused by adding cognitive load.

### Vision

Electrodermal Response has been shown to be greater when peripheral vision is occluded in stance (Davis et al., 2009), and in a quiet stance at the edge of the support surface, the EDL in trials with the eyes closed is lower than that with the eyes opened (Sibley et al., 2007). In spite of those previous findings, the influence of vision on the mental response induced by the perturbation in stance has not been elucidated. We hypothesized that EDR induced by perturbation in stance is greater when vision is occluded, because perturbation in stance is more threatening when vision is available (Hypothesis 5). Visual occlusion did not induce a significant change in EDR amplitude or latency and thus this finding did not support this hypothesis.

The pelvis displacement during the forward perturbation was greater when vision was present. According to studies in blind patients, the long-term absence of vision enhanced the reliance of voluntary motor control on proprioception (Yoshimura et al., 2010; Ozdemir et al., 2013). Those findings are likely explained by inter-modal reweighting between vision and proprioception (Logan et al., 2014), expressed as an increase in the role of the proprioception when vision is lost. Accordingly, greater pelvis sway induced by perturbation with vision may be explained by a view that the contribution of the proprioception, which plays a role in reducing body sway, is lesser when vision is available.

### Limitations

The sample size of the present study was determined based on the sample size of the previous studies that investigated the effect of the postural perturbation on the EDR (Sibley et al., 2007, 2008, 2009, 2010a,b). We did not conduct the power analysis to determine the sample size. Thus, we cannot rule out type II error for the negative findings. Sampling rate of the signals from the accelerator was 1 kHz. In this case, Nyquist frequency is 500Hz (Nyquist, 1928). However, high-cut filter of the accelerometer was 1 kHz in the present study. Thus, we cannot rule out the aliasing of the signals from the accelerometer.

## Conclusion

Translation of the support surface in stance induced one- or two-peak EDR 1–1.5 s after the translation. The first component of the two-peak EDR was an additional component preceding one-peak EDR. Across-trial variations in EDR size was positively correlated with across-trial variations in fear. These findings indicate that perturbation-induced EDR in stance sometimes represents multiple mental responses that include perturbation-induced fear of fall. The perturbation-induced EDR in stance was influenced by the perturbation’s characteristics, indicating that perturbation-induced EDR in stance is a task-specific response. The EDR appeared earlier when the perturbation was self-triggered or when the participants prepared to answer a question, indicating that adding cognitive load induces earlier mental responses to perturbation in stance.

## Data Availability Statement

The raw data supporting the conclusions of this article will be made available by the authors, without undue reservation.

## Ethics Statement

The studies involving human participants were reviewed and approved by Graduate School of Comprehensive Rehabilitation, Osaka Prefecture University Committee on Research Ethics. The patients/participants provided their written informed consent to participate in this study.

## Author Contributions

TK and KH planned the study, conducted the experiment, performed the data analysis, and wrote the manuscript. HO conducted the experiment. All authors contributed to the article and approved the submitted version.

## Conflict of Interest

The authors declare that the research was conducted in the absence of any commercial or financial relationships that could be construed as a potential conflict of interest.

## Publisher’s Note

All claims expressed in this article are solely those of the authors and do not necessarily represent those of their affiliated organizations, or those of the publisher, the editors and the reviewers. Any product that may be evaluated in this article, or claim that may be made by its manufacturer, is not guaranteed or endorsed by the publisher.
